# Poststroke Cognitive Impairment Research Progress on Application of Brain-Computer Interface

**DOI:** 10.1155/2022/9935192

**Published:** 2022-02-07

**Authors:** Xiaowei Sun, Mingyue Li, Quan Li, Hongna Yin, Xicheng Jiang, Hongtao Li, Zhongren Sun, Tiansong Yang

**Affiliations:** ^1^Heilongjiang University of Chinese Medicine, 24 Heping Road, Xiangfang District, Harbin, China 150036; ^2^First Affiliated Hospital, Heilongjiang University of Chinese Medicine, 26 Heping Road, Xiangfang District, Harbin, China 150036; ^3^Second Affiliated Hospital, Heilongjiang University of Chinese Medicine, 411 Guogeli Road, Nangang District, Harbin, China 150000; ^4^Department of Rehabilitation Medicine, Shenzhen People's Hospital, Second Clinical Medical College of Jinan University, Shenzhen, China 518120

## Abstract

Brain-computer interfaces (BCIs), a new type of rehabilitation technology, pick up nerve cell signals, identify and classify their activities, and convert them into computer-recognized instructions. This technique has been widely used in the rehabilitation of stroke patients in recent years and appears to promote motor function recovery after stroke. At present, the application of BCI in poststroke cognitive impairment is increasing, which is a common complication that also affects the rehabilitation process. This paper reviews the promise and potential drawbacks of using BCI to treat poststroke cognitive impairment, providing a solid theoretical basis for the application of BCI in this area.

## 1. Introduction

Strokes rank first among long-term disabling diseases [1]. Poststroke cognitive impairment (PSCI) is one of the most common residual symptoms of strokes. In a recent review and meta-analysis of hospital-based studies, PSCI is reported to be 53.4% after stroke. Results from the STROKOG consortium showed different domains of cognitive impairment in 30–35% of patients a short time after a stroke [2]. It not only affects the quality of life of stroke patients but also places a heavy burden on society and the economy. An important feature of PSCI is that it is preventable and treatable [3], so it is important to explore how to improve cognitive function after strokes using modern neurorehabilitation techniques. Brain-computer interface (BCI), as a new rehabilitation technology, not only can be used to evaluate the efficacy of cognitive impairment after strokes [4] but may also be applied to the rehabilitation of cognitive ability. This is of great significance for the early diagnosis and treatment of cognitive impairment after stroke, and for preventing mild cognitive impairment from developing into vascular dementia or other diseases.

## 2. Overview of PSCI

Poststroke cognitive impairment (PSCI) is one of the major complications of strokes and refers to a series of syndromes from mild cognitive impairment to dementia caused by strokes [5]. PSCI is common in all stroke subtypes, and even patients with transient ischemic attacks (TIA) are at risk of developing cognitive impairments and dementia [6]. PSCI can affect multiple cognitive domains, including executive functioning, memory, attention, language, and visuospatial abilities [7], although executive dysfunction and memory impairment are the most common [8]. Recent studies have found that within a year after stroke occurrence, as many as 53.4% of patients could show cognitive impairments [9], and the proportion of mild cognitive impairment could be as high as 80%. Additionally, more than 7% of PSCI patients could develop dementia [10].

The type of stroke lesions, reperfusion status, brain compliance, and nutritional status of stroke patients are all related to the rehabilitation of stroke-related cognitive impairment [11]. Additionally, in recent years, functional neuroimaging studies have found that cognitive impairment after stroke is also associated with lesions in distal brain regions and changes in brain network connectivity [12]. Dacosta-Aguayo et al. used the resting-state functional magnetic resonance imaging (RS-fMRI) technology to study these phenomena and found that PSCI patients had reduced functional connectivity [13]. Another large study of heterogeneous stroke patients also found that damage to certain brain regions may lead to disturbances in brain networks and a variety of cognitive symptoms [14]. Through neuroimaging studies, Jaywant et al. found that poststroke executive dysfunction was related to changes in resting-state functional connectivity. Overconnectivity of the cognitive control network and reduced connectivity of the transhemispheric frontal and parietal networks were closely related to poststroke executive function. Therefore, cognitive training that targets brain networks is also helpful for treating executive dysfunction following stroke [15]. In addition, nonverbal cognitive impairment in patients with aphasia after stroke has been shown to be associated with extensive destruction of white matter microstructure integrity, wherein uncinate fascicle (UF) damage is closely related to spatial perception (SP) and motor practice (MP) deficits [16].

PSCI evaluation usually employs neuropsychological tests such as the Montreal Cognitive Assessment (MoCA) and Mini-Mental State Examination (MMSE). However, these scales usually rely on subjective judgment and do not contain domain-specific cognitive assessment information (i.e., they do not measure reading or writing abilities). As a result, test results may not be accurate for PSCI patients [17]. Additionally, there is no effective treatment strategy for PSCI-related cognitive decline. Currently, drug therapy and rehabilitation therapy are the main clinical treatments for PSCI. The purpose of drug therapy is mainly to control the risk factors related to cerebrovascular disease, to improve the main symptoms of cognitive impairment and accompanying mental symptoms such as depression and anxiety, and to delay the progression of the disease [18]. However, there are many side effects associated with drug therapy. Acupuncture has also been used as a complementary and alternative therapy for patients who do not respond well to drug therapy [19]. Acupuncture can also improve the cognitive functioning of patients with poststroke cognitive impairment but no dementia (PSCIND) and reduce the chance of developing PSD [20].

Rehabilitation treatment mainly involves cognitive rehabilitation, including relearning previously learned knowledge or gaining new knowledge—which causes functional changes and enhances cognitive functioning. Cognitive rehabilitation mainly includes traditional cognitive retraining and cognitive enhancement with the application of high-tech equipment. Noninvasive brain stimulation (NIBS) has been used for the treatment of PSCI, but the selection of stimulation site, stimulation parameters, and mechanisms need further study [21]. In addition, adaptive conjunctive cognitive training (ACCT) also has positive effects on PSCI patients' attention and spatial awareness and reduces depression symptoms [22]. Finally, artificial intelligence, including neurocognitive robots [23], and computer-assisted cognitive training have also been employed to improve cognitive impairment in stroke patients [24]. Current cognitive rehabilitation treatments are often only used for defects in cognitive field training, and PSCI patients tend to show higher rates of cognitive defects. Further, the patient's mental health also plays a role in reducing cognitive impairment [25], so multimodal assessment and rehabilitation should be used for PSCI patients. As an emerging technology, BCI uses brain neural activity as input, employs mathematical algorithms to decode neural signals, and converts intentions or decisions into commands for external machines such as computers. These computers can then be used to monitor subjects' mental state or to improve cognitive abilities. In recent years, BCI has been employed to treat a variety of neurological diseases. Further, researchers have applied different BCI schemes to improve PSCI, which is of great importance for treating PSCI-related cognitive impairments, and for the prevention of vascular dementia and other diseases.

## 3. Overview of BCI

### 3.1. Development of BCI

As early as 1969, Rosenfeld et al. [26] detected modulated visual and auditory responses in animals. Later, inspired by the adjustable nature of brain activity, Professor Vidal from the University of California, Los Angeles, created BCI to realize a technology for reading brain signals [27] and first proposed the idea of using operant conditioning to control computers. It was not until 1977 that Pfurtscheller and Aranibar found through experiments that subjects could change the frequency band power of EEG alpha (8-12 Hz) and beta (12-25 Hz) signals in the motor region of the brain by moving or imaginatively moving certain body parts and that changes would occur both at the beginning of movement and during the migration process. This marked the formation of the first human biofeedback BCI [28].

### 3.2. Construction of BCI System

**Figure 1:**
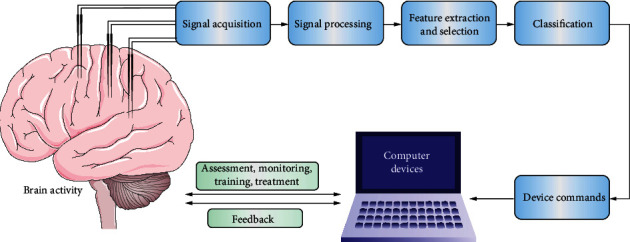
Basic layout and process of a BCI system.

In contrast to the conventional brain information output pathway, BCI is a new communication and control system that connects the brain or nervous system to any device capable of processing or computing. BCI can be controlled by various signals sent by the brain. These electrical, magnetic, or metabolic activity signals can then be further amplified, filtered, decoded, and translated into signals to control external devices. A complete BCI system usually consists of signal acquisition, signal processing, feature extraction and selection, signal classification, external control, and user feedback. Each of these parts has a variety of developed methods, and a heterogeneous combination of different approaches allows the BCI to be customized to meet specific disease needs (Figure 1).

#### 3.2.1. Signal Acquisition and Processing

BCI can be divided into two types—noninvasive and invasive—depending on signal acquisition mode. Noninvasive BCI is a safer, more convenient, and noninvasive technique to obtain human brain signals directly from the scalp. Among noninvasive BCIs, EEG is the most commonly used signal acquisition method. These BCI systems are divided into exogenous BCI and endogenous BCI according to the source of EEG stimulation. Exogenous BCI refers to EEG signal patterns induced by external stimuli, such as event-related potentials (ERPs) [29], auditory steady-state responses (ASSR) [30], steady-state visual evoked potentials (SSVEPs), and P300 [31]. Hwang et al. introduced a novel BCI mode based on ERP-BCI for patients with complex eye dysfunction, which does not rely on gaze function and can complete visual stimulation under closed eyes [32]. Hill et al. are developing new BCI systems to make previously employed ASSR stimulations more natural and intuitive [33]. Jiang et al. developed new BCI systems based on EEG of the event-related potential-neurofeedback-brain-computer interface (ERP-NF-BCI) platform used for training. During the training process, subjects' brain electrical signals were captured using a wireless EEG headset. At any given time, subjects were instructed to direct their attention to tasks related to a stimulus or to ignore an irrelevant stimulus. Patients trained the EPR-NF-BCI system to provide positive feedback and improve target visual stimulus attention abilities [34]. Among all of these systems, BCI based on P300 is the most popular because it has high classification accuracy and a fast information transfer rate (ITR). In addition, the hold-release function developed by Alcaide-Aguirre et al. allows for faster (6-16 times) and more continuous control of P300-BCI [35], and changes in patients' visual and auditory senses affect its performance [36]. However, when SSVEP-BC-based visual stimulation is applied in elderly patients, eye fatigue is common, and epileptic seizures are sometimes induced. Another BCI system is based on the endogenous BCI paradigm, which uses self-modulating EEG signal patterns without external stimulation, such as sensorimotor rhythms (SMRs) [37], slow cortical potentials (SCPs) [38], and signals generated by imagining motor movements without the need for actual muscle movement.

Magnetoencephalography (MEG) has many advantages over electroencephalography, such as its ability to record gamma signals from the cortical sulcus and from higher wavelengths. However, MEG is rarely applied because it needs to use expensive superconducting materials [39]. Functional magnetic resonance imaging (fMRI) can also reflect the activity of neurons by measuring blood flow signals. Sulzer et al. found that subjects were able to control blood oxygenation level-dependent (BOLD) signals in specific brain regions and that the BOLD signals were related to low and high-frequency field potentials in BCIs [40]. However, real-time fMRI has poor temporal resolution and a high price, so it is not applicable to BCIs. Functional near-infrared spectroscopy (FNIRS), by contrast, is cheaper and more portable. However, the signal-to-noise ratio (SNR) and spatiotemporal resolution of FNIRS are also limited, and the communication rate is even lower than that of EEG-based BCI [41]. For invasive signals, signals on the pia mater surface (also known as electrocorticogram (ECoG)), signals on the dura mater surface (epidural field potentials (EFPs)), or signals in the cortex (sharp waves or local field potentials (LFPs)) are usually used [42]. For example, Vansteensel et al. designed an ECoG-BCI-based cortical intracortical brain-computer interface (IBCI) for typing and playing games [43]. Moses et al. used a high-density cortical electrogram to realize real-time decoding of the superior temporal gyrus during an auditory exercise [44]. Research by Pandarinath et al. further showed that IBCI could provide high communication rates [45]. In addition, biofeedback BCIs based on LFPs may also be able to obtain more reliable neural signals for corresponding regulation [46]. Compared with noninvasive signals, invasive signals have a higher SNR, higher communication rate, and higher temporal and spatial resolution. In order to improve BCI performance, a series of problems must be overcome—including low classification accuracy, small degrees of freedom, and a steep learning curve for understanding how to operate BCI. Hybrid brain-computer interfaces (hBCIs) and multimode BCIs have been developed for rehabilitation that attempt to solve these problems—including P300 and SSVEP, P300 and motor imagery (MI), EEG and eye movement (EOG), EEG and electromyography (EMG), and EEG and electrocardiogram (ECG) setups [47]. For example, Fazli et al. [48] developed a multimodal BCI that combined EEG and near-infrared spectroscopy (NIRS) and improved the signal classification accuracy for 90% of participants. In addition, Wang and Jung also proposed a collaborative BCI that integrates information from multiple users. Compared with single-user BCIs, this system effectively integrates the brain activities of a group of people, which vastly improves the overall performance [49]. Multimodal BCI has high sensitivity and specificity and is resistant to ambient noise. The era started with noninvasive brain-computer interfaces (BCIs), based on electroencephalography (EEG) [50]. Noninvasive BCI systems have one advantage over the invasive methods, as they do not require any surgical intervention, and their implementation is neither difficult nor risky [51]. Their nature of being noninvasive has made this technique popular. The application potential is vast and ranges from clinical to home-entertainment applications, such as the popular and inexpensive customer-grade EEG headsets [52]. There is still a drive toward a more cost-effective, smaller, portable, and efficient device in medicine especially in the neuroscience field [53, 54]. The future direction also involves a combination of noninvasive BCIs, coupled with augmented reality (AR) systems. Future trends in the development of the BCI systems are probably strongly correlated with the development of intelligent algorithms for the analysis of biomedical data and the systems with a reduced number of channels [55]. Therefore, noninvasive BCIs and hBCIs based on EEG show the most promise for application in neurological rehabilitation (Table 1).

#### 3.2.2. Feature Extraction and Feature Classification

Frequency bands recorded by EEG can be identified and used to express the patient's intention after signal transformation and classification. Therefore, effective extraction of EEG signal characteristics and accurate classification are two of the important steps for any BCI system. Kober et al. used an EEG Fast-Fourier Transform (FFT) feature extraction method to detect the effects of neurofeedback (NF) training on memory improvement following stroke [56]. Ieracitano et al. used the continuous wavelet transform (CWT) to develop a new automatic classification method of EEG records based on multimodal machine learning for the detection of patients with mild cognitive impairment (MCI) or Alzheimer's disease (AD) [57]. Based on P300-BCI, Onishi and Natsume improved the linear discriminant analysis (LDA) feature classifier into an integrated stepwise linear discriminant analysis (SWLDA) classifier with overlapping partitions, which significantly improved BCI performance [58]. Chen et al. [59] proposed a BCI based on the SSVEP innovative coding method, which uses a relatively large frequency flicker (10, 12, and 15 Hz) stimulation of brightness, and low-frequency alternating (0.5, 1 Hz) color modulation, to induce intermodulation frequencies at the same time. This increases the number of single frequency flashes and allows the system to have a classification accuracy rate of 93.83% and ITR of 33.90 bits/minute. Some new EEG feature extraction and classification have also recently emerged. For example, the sparse Bayesian learning [60] has been used to predict the behavior or cognitive state of subjects. Deep learning algorithms [61] are also used to extract multisource EEG signals and carry out high-precision classification. In addition, the use of complex networks is a new method which can be used to analyze the structural changes of brain networks in patients with nervous system diseases and to reveal the relationship between brain functional patterns and disease progression. In addition, the Granger causality methods are also used to evaluate brain connectivity [62]. These newly optimized feature extraction and classification methods continue to improve BCI performance, which provides a good technical foundation for the rehabilitation of cognitive impairment after stroke.

#### 3.2.3. External Control and User Feedback

External control involves the processing of collected signals into digital commands using a combination of filtering, transformation, and classification algorithms, and then receipt of those signals by effectors. Any device that can be programmed to receive functional commands can be used in the rehabilitation of cognitive impairment after stroke. However, fully functional BCI systems provide feedback only online to the user after the effectors receive instructions. Feedback methods include visual, auditory, and tactile sources, among which visual feedback is most common [63]. There is also neurofeedback (NF), which is a special form of feedback based on EEG-BCI. During NF, the application interface displays brain activity intuitively and in real time to the user. The user can then self-regulate brain functioning according to the feedback and make it return to a normal state. NF techniques are usually based on EEG or RT-fMRI. NF training based on EEG signals involves repeated tasks and has been shown to improve attention, executive function, and memory [64]. Robineau et al. showed that, in six stroke patients, RT-fMRI-based NF improved visual stimulation awareness [65]. In addition, NF training can also regulate sensory motor rhythm (SMR) in stroke patients and healthy elderly people and is associated with significant improvements in behavior and memory during nonverbal learning tasks [66]. In addition, some BCI systems synchronize neural activity with feedback devices to create closed-loop multimodal feedback, enhance the Hebbian plasticity, and help restore motor function. Further, closed-loop BCI systems can also be used as biofeedback platforms to improve and enhance individual cognitive ability. New settings, including multiplayer collaboration and EEG-NF in virtual environments and videogames, are also constantly being developed in order to make BCI more powerful, exciting, and challenging [67].

### 3.3. The Mechanism of BCI Promoting Cognitive Rehabilitation after Stroke

#### 3.3.1. Promote Neural Remodeling

The human brain performs complex cognitive functions such as learning, memory, and emotion processing through the normal activity of nerve cells. When the body's normal neural pathways are injured, BCI can serve as part of the injury “bypass.” BCI connected directly to an external control device, or BCI used in tandem with other techniques (i.e., functional electrical stimulation (FES) and virtual reality (VR)), has been shown to promote functional recovery [68–70]. Nie and Yang showed that MI promoted functional neuron remodeling, increased the expression of scaffold proteins and regulatory proteins, enhanced synaptic plasticity, and promoted learning and memory-related cognitive functions [71]. Ortiz et al. also confirmed that a new type of BCI based on gamma bands could enhance neuroplasticity, promote cognitive and motor rehabilitation, improve the operating accuracy of exoskeleton control, and improve attention levels during gait walking [72]. Kleih et al. also proposed that BCI could enhance the plasticity of neurons by activating language circuits and thus promote the recovery of language functions in patients with aphasia after stroke [73]. fMRI and diffusion tensor imaging further confirmed the neuroplasticity of stroke patients after BCI therapy [74]. On this basis, Zuo et al. [75] proposed a hybrid BCI system that involves both MI and P300. In this program, 12 healthy subjects were asked to imagine writing Chinese characters in a specific order. Results showed that the recognition accuracy of patients exposed to mixed BCI was significantly higher than that of those exposed to single P300 (*P* < 0.05) or MI (*P* < 0.01) BCI alone.

#### 3.3.2. Promote Neural Network Recovery and Enhance Brain Connectivity

Changes in brain network connectivity patterns are highly predictive of cognitive performance. Cassidy and Cramer have argued that all cognitive rehabilitation-related neuronal remodeling phenomena are related to changes in brain network structures [76]. Fodor et al. used 128-channel EEG to study event-related synchronic potentials (ERS) in 17 patients with mild cognitive impairment (MCI) and 21 healthy controls during the Sternberg working memory task and found that event-related synchronic (ERS) potentials in *α* and *β* bands were significantly reduced in patients with MCI, indicating early impairment of neural networks related to working memory [77]. Toppi et al. also estimated the brain connectivity of patients with memory deficiencies after stroke based on the EG-BCI-NF training method and found that improvement of memory function was associated with increases in the predensity index of brain connectivity and the left temporal alpha band, indicating that memory improvements are related to brain network functioning [78]. Taken together, all of these studies provide strong evidence for the utility of BCI for clinical treatment. Zhang et al. also found changes in brain connectivity related to the use of a motor imagination brain-computer interface (MI-BCI) in the field of poststroke rehabilitation. The results showed that right ventral internal parietal sulcus degree centrality (DC), left parietal lobe eigenvector centrality (EC) and cortical thickness (CT), and right dorsolateral prefrontal cortex CT were significantly correlated with MI-BCI. In addition, analysis of subjects' working characteristics and machine learning classification found that the EC and CT could effectively predict the low intelligent users from the high intelligent users with an accuracy of 83.3%, indicating that BCI based on brain connectivity can also be used for cognitive assessments [79].

## 4. Application of BCI in PSCI

BCI was first used in stroke rehabilitation treatment in 2009 [80].BCI can not only detect brain activity—it can also be used for cognitive assessment and training for patients with cognitive impairment, help patients express their intentions, address cognitive and memory impairments, and promote communication. BCI technology has broad development prospects for improving cognitive functioning, patient autonomy, and quality of life. The range of applications of BCI in PSCI is shown in Table 2.

Table 2 contains a total of 16 articles, of which 2 articles are on BCI assessment, 4 articles focus on the training of cognitive function by BCI, and 8 articles about the treatment of cognitive impairment by BCI. Park et al. [82] used BCI to assess cognitive engagement after stroke, while Shukin et al. [90] used BCI to evaluate the efficacy of poststroke rehabilitation training with BCI. Through different methods of BCI cognitive training [81, 83, 85, 91], there are significant improvements in multiple domains of cognitive impairment, including executive ability [83, 89], language ability [84], attention [87, 92], visuospatial ability [91, 93], and memory [85, 94–96]. One of the other two articles showed that mental fatigue state influenced the assessment and performance of BCI [88], while the others showed that brain-computer interface rehabilitation training was ineffective in patients after stroke. See the application below for specific analysis.

### 4.1. Assessment of BCI for PSCI

With the development of new intelligent rehabilitation technologies, BCI systems can also provide more objective and accurate neuropsychological assessments for PSCI patients. Zhang et al. designed a cognitive functioning system for MCI screening using BCI technology. The results from the new functional assessment system were highly correlated with the traditional Montreal cognitive assessment system (*r* = 0.83) [79].

Park et al. used EEG to evaluate the cognitive engagement of 11 patients with chronic stroke while performing motor tasks and observed that active motor tasks induced greater event-related desynchronization (ERD) in the bilateral motor cortices and supplementary motor area (SMA) than did passive motor tasks [82]. In addition, Lyukmanov et al. used a BCI-based system to conduct neuropsychological tests on 55 patients with motor disorders after their first stroke who were undergoing rehabilitation training. The Fugl-Meyer assessment (FMA) and action research arm test (ARAT) were used to detect the severity of motor impairment and arm paralysis after stroke in the control group. The BCI group received BCI-based neuropsychological tests and motor imagination training that incorporated exoskeleton feedback under the control of BCI. At the end of the evaluation, both groups showed improvements in ARAT and FMA (parts A-D, H, and I), but only the BCI group showed improvements in ARAT's grasp score (*P* = 0.012), pinch score (*P* = 0.012), gross movement score (*P* = 0.002). Certain neuropsychological tests (i.e., the Taylor figure test, choice response test, and head test) were significantly correlated with online accuracy. These results suggest that increasing the level of BCI control in exoskeleton-assisted physical therapy can significantly improve rehabilitation effects after stroke [97]. At the same time, BCI can also monitor the global attention level related to task processes, and monitoring the changes in attention during BCI training can ensure better focus on the current task [98].

BCI can also be used to evaluate efficacy. Shukin et al. [90] used cognitive P300-evoked potentials and a diagnostic scale to evaluate treatment dynamics in patients with cognitive impairment of chronic cerebral ischemia. Patients with chronic cerebral ischemia aged were divided into a treatment group and a control group and were treated with cytoflavin and methyl succinate hydroxypyridine, respectively. During the treatment period, the neurophysiological parameters of both groups improved, especially in the patients treated with cytoflavin. The amplitude of P300 in the left hemisphere was 9.21 (8.36, 10.11)~12.41 (10.23, 13.37) *μ*V, which was a 1.3-fold increase. The right hemisphere amplitude was 6.48 (5.26, 7.35) to 11.04 (9.29, 12.18) *μ*V, a 1.7-fold increase.

BCI can also be used to monitor physiological changes in stroke patients. J Wilson et al. [99] reported that a platform based on BCI COSBID-M3 multimode monitoring for stroke and other neurological diseases could monitor cortical functioning and pathology in real time during surgeries. In summary, BCI has been widely applied to assess cognitive functioning, but the single mode BCI also has some drawbacks, including unreliable data, long length of assessments, and fatigue. Therefore, the application of multimodal BCI and modified BCI for multiple cognitive tests may be more effective for comprehensive clinical assessments.

### 4.2. Training of BCI for PSCI

The application of BCI training for the rehabilitation of limb motor function after stroke is developing rapidly. Cognitive training is another focus of neurorehabilitation research. Kruse et al. conducted a meta-analysis on the influence of BCI training on the recovery of brain function in patients with strokes and concluded that BCI training could enhance recovery [100]. Lee et al. have also shown that BCI training can improve attention, visuospatial abilities, and memory in older adults. They are also developing a larger BCI cognitive training intervention trial for the cognitive assessment of patients with early dementia [101].

Neurofeedback training (NFT) and motor imagination (MI) training are two common methods of BCI cognitive function training. Cho et al. [91] found that NFT showed a significant increase in attention and visual perception over traditional rehabilitation training. They also showed significant changes in EEG-detected beta values, indicating that NFT can actually improve cognitive performance. BCI devices also can monitor NFT influences on memory function. One example is the work of Silvia Erika Kober, on memory defects in stroke patients. Before NFT, EEGs showed that the left brain artery ischemic stroke patients' hemispheres had pathological delta (0.5-4 Hz) and highest alpha (10-12 Hz) frequencies. After NFT, the EEG showed more standard frequency topography on both sides. Memory tests on patients with bilateral subarachnoid hemorrhage revealed significant improvement in both short-term and long-term memory and slight improvement in working memory, following NFT. Patients with left cerebral artery ischemic strokes had significant improvements in long-term memory after NFT [85]. Ruiz et al. [102] found that conscious control of the anterior insula increased cognitive flexibility in a facial emotion recognition task in healthy elderly people through real-time BCI-NFT, further demonstrating the effectiveness of BCI in cognitive enhancement and training.

Gomez-Pilar et al. [103] showed that NFT based on MI-BCI could enhance cognitive function in elderly patients. In this study, 63 subjects were recruited—31 in the NFT group and 32 in a control group that did not receive training. Subjects were asked to practice five tasks of increasing difficulty over and over again before giving neural feedback through a motion visual-controlled, moving-on-screen program. Cognitive test results showed that, after five NFT sessions, four measures of cognitive function (visuospatial, language, memory, and intelligence) improved significantly (*P* < 0.01). Thus, repeated BCI training may promote neural plasticity by repeatedly stimulating the parts of the brain involved in cognitive processing. In addition, studies have shown that MI can promote neural plasticity [104]. Yan et al. [81] used EEG to study 11 patients with left hemisphere ischemic cerebral apoplexy and found that, after MI training, cognitive changes occurred. MI training has been widely used to promote functional rehabilitation after stroke. In addition, BCI can provide real-time, quantitative monitoring of brain function in conjunction with MI training. Pichiorri et al. [83] studied 28 patients with subacute severe cerebral apoplexy dyskinesia and used BCI to support MI training. They found that, compared with patients who underwent unsupported MI training, the BCI training upper-limb Fugl-Meyer assessments improved significantly (*P* < 0.03), sensorimotor alpha and beta bands were more highly synchronized, and resting ipsilateral brain connectivity within the same bands increased significantly (*P* < 0.05).

BCI training is also affected by language, gender, and other conditions. BCI versions and language settings may also be different. However, even when employed in patients who speak different languages, there appear to be no significant differences in BCI effectiveness. Lee et al. [105] used EEG-based BCI in 32 English-speaking and 39 Chinese-speaking elderly patients and found that cognitive abilities were improved in both groups. However, gender has a moderating effect on BCI. For example, Yeo et al. [106] applied a BRAINMEM training system to improve cognitive functions, such as attention, working memory, and delayed recall in the elderly, and found that there was no significant difference in overall cognitive performance between the training group and the nontraining group after treatment. In men, however, the intervention group performed better than the control group (*P* = 0.046).

### 4.3. Treatment of PSCI by BCI

The treatment of PSCI by BCI mainly manifests in improved executive function, attention, memory, language, and visuospatial abilities.

#### 4.3.1. Executive Function

Due to executive dysfunction after stroke, patients may be uncoordinated and/or experience judgment errors while driving. However, BCI may remedy these problems [107]. Chung et al. used brain-computer interface-controlled functional electrical stimulation (BCI-FES) rehabilitation techniques in patients with chronic hemiplegic stroke and found that the training significantly approved their ability to walk after stroke and that these differences were also significantly increased compared to patients who experienced FES rehabilitation only [89].

#### 4.3.2. Memory Function

Memory is a cortical function that preserves information and past experiences and helps people acquire new skills and learn new information. Memory can also be divided into short-term and long-term memory. Due to severe functional damages and memory loss in stroke patients, early memory deficit intervention may help prevent the disease from progressing to Alzheimer's disease or vascular dementia [108].

BCI technology has been shown to improve memory, attention, and consciousness in older people with cognitive impairments. Lee et al. [109] used EEG-BCI in 31 healthy elderly patients and measured cognitive improvements using cognitive ability tests, card matching games, and other memory and attention tasks. The results showed significant improvements in immediate memory (*P* = 0.038), delayed memory (*P* < 0.001), and concentration (*P* = 0.039) scores. In addition, NF therapy based on EEG can also regulate the brain activity of stroke patients and help restore memory functions. Reichert et al. [94] applied NFT to basilar artery thrombosis stroke patients and found that sensorimotor rhythm neurofeedback (SMR-NF) training positively affected memory functioning. Prior to starting NFT, patients presented with short- and long-term memory deficits (*T* − scores < 40). After SMR-NF, the performance of various memory functions was better than expected.

Toppi et al. [95] studied the effects of a BCI closed-loop neurofeedback intervention scheme. Two stroke patients (patient A, female, 70 years, right hemisphere stroke lesion; and patient B, male, 20 years old, left hemisphere stroke) underwent 10 sessions of SMR-NF training to attempt to address memory impairments. Neuropsychological tests showed that, after NF training, one of the patients' performance accuracy on the Sternberg memory task was significantly increased and reaction time was significantly decreased (*P* < 0.05). Auditory memory and visuospatial short-term memory impairments were also significantly improved after training (*P* < 0.05). Finally, the Rey auditory verbal learning test (RAVLT) and Corsi block tapping test (CBTT) equivalent scores increased from 1 to 3 and 4, respectively. In addition, in an attempt to enhance memory function, Burke et al. [110] used intracranial electroencephalography (iEEG) in neurosurgery patients to detect *θ* wave and *α* wave oscillations associated with optimal memory encoding. They aimed to trigger the occurrence of relevant memory encoding waveforms during the process of recall. This was the first time that iEEG was used to enhance episodic memory in BCI. Kober et al. [96] studied the SMR of 17 stroke patients (11 experienced 12 to 15 Hz and 6 experienced upper alpha frequencies of 10-12 Hz). Results demonstrated that patients in the SMR group showed improvements in short-term memory performance, and working memory performance improved in patients in the upper frequency group. Thus, the effects of NFT in stroke patients were better than those of traditional cognitive training.

#### 4.3.3. Attention

Attention is the core component of cognitive ability. Inattention damages memory and behavioral performances. Most cognitive training therefore seeks to improve attention abilities. Attention is controlled by a network of interconnected cortical regions, including the frontal visual field, the parietal area, some subcortical structures, the superior colliculus, and oculomotor muscles [111]. The frontal cortex region, which plays a key role in attentional control, can be detected by EEG or LFPs as an attention marker due to synchronous neuronal activity [112]. Using EEG-NF-BCI, which measures neural signals and is used to enhance attention and cognitive performance, patients can observe a graphical representation of their brain activity, which is also self-regulated by computer processing into an optimal state. This method has been used to treat a variety of neuropsychological disorders, including PSCI [92]. Foong et al. [88] conducted EEG-based MI-BCI in 11 stroke patients (mean age 55.2 ± 11.0 years) using visual feedback and found significant changes in *β*-power and EEG signals in frontal and central brain regions when fatigue occurred during the test, indicating that mental fatigue may affect BCI performance to a certain extent. In addition, BCI rehabilitation requires the ability to focus on screens for a long time, so cognitive impairments such as attention deficits in stroke patients may also have an impact on BCI performance. For example, in the P300-BCI system, decreased attention levels and the high working memory loads can result in the ERP signal within the P300 system having low amplitude and long latency period. However, in the nonvisual BCI mode that is based on P300, increases in P300 amplitude may indicate the improvement of attention after training [113]. In recent years, because of new developments in brain imaging and BCI technology, real-time fMRI closed-loop training has successfully improved visual attention and behavioral performance [114]. Thus, while BCI can improve attention, the degree of attention can also affect the performance of BCI.

#### 4.3.4. Language Ability

Another important application of BCI in the recovery of cognitive function is the rehabilitation of speech ability in stroke patients. Flowers et al. reported that more than 30% of all stroke survivors are affected by speech impediments [115]. Compared with stroke patients without aphasia, poststroke aphasia (PSA) patients tend to have more extensive and severe nonverbal cognitive impairments. Among these patients, patients with nonfluent aphasia tend to have more severe disorientation and spatial perception impairments than patients with fluent aphasia [116]. Nolfe et al. [117] suggested that P300 may predict aphasia recovery, and studies have found that the amplitude of P300 decreases in aphasia patients. On this basis, Kleih [84] used the P300-BCI spelling system to assess language functioning in patients with poststroke aphasia. The experiment included five patients with aphasia after stroke. The researchers applied EEG-P300, and ERP during spelling and reading practice, and found that four patients with aphasia after stroke who were initially unable to use the visual P300 could successfully communicate using the P300-based BCI speller with 100% accuracy. One patient who was dyslexic following a stroke was able to read 14-letter words, up from 9-letter words, after BCI training. In addition, the accuracy of spelling and reading improved when attention was focused. The P300 amplitude and attention performance test (German: Testbatterie zur Aufmerksamkeitsprüfung (TAP)) was improved after training in two patients with aphasia after stroke, suggesting that the visual P300-BCI spelling system could be used for language training and could be used to judge cognitive abilities after stroke. However, unlike English words, Chinese characters are usually written with two-dimensional structures. Thus, Han et al. [118] developed a novel Chinese character writing robot controlled by BCI, which used the mixed features of P300 and SSVEP to effectively encode a large instruction set, decode the combined features using take related component analysis, and generate efficient writing of both Chinese characters and English letters. The average accuracy was 87.23%, and the maximum accuracy was 100%. The corresponding information transmission rates were 56.85 bit/min and 71.10 bit/min, respectively. In addition, BCI can also identify EEG signals sent through the BCI and then transmit them to the corresponding receptive brain region as new incoming information. Thus, BCI can facilitate two-way dialogue between two people who cannot communicate [119, 120]. All these factors suggest promise for the application of BCI to improve language abilities in stroke patients.

#### 4.3.5. Visuospatial Ability

BCI can also be used to improve visuospatial abilities. Tonin et al. [86] have shown that BCI can improve laterally dominant attentional visuospatial deficits. By using covert visual spatial attention- (CVSA-) BCI in three patients with left spatial neglect (SN) stroke, they found that the patients could control CVSA-BCI with accuracy rates above 50%. Behavioral RTs were also decreased in two patients (*P* < 0.01). Further, the *α*-peak loss ratio was significantly decreased (*P* < 0.01), and the asymmetry between hemispheres in the parietooccipital region showed significant improvements (*P* < 0.05). In stroke patients, FC between the right hemispheres was significantly increased, suggesting that CVSA-BCI may help enhance neuroplasticity, reduce the imbalance between hemispheres, increase the connectivity between hemispheres, improve attention, and remedy visuospatial defects.

The BCI multimodal analysis can also predict the cognitive processing depth of visual imagination, memory, language, and other task domains [93]. Hreha et al. [121] followed 1439 stroke patients and used a regression model to observe the relationship between visual acuity and changes in cognitive function. They found that overall visual acuity was associated with a significant decrease in baseline cognitive function. Further, visual impairment (VI) was not associated with rates of cognitive decline.

Kotov et al. [87] studied the effects of multimode BCI stimulation on cognitive function recovery in patients with strokes. A total of 44 patients were examined and treated between 2 months and 2 years after stroke. After treatment, memory, attention, and visual spatial abilities of patients in the treatment group showed significant improvements compared with those in the control group. Thus, multimode and multichannel BCI may help activate neural plasticity, improve the relationship between hemispheres, and promote the recovery of cognitive function in patients with stroke.

In conclusion, BCI has been shown to have many positive effects on PSCI patients, but its long-term efficacy may need to be further verified. It is also worth noting that some studies have shown that BCI is ineffective in improving PSCI. Sebastián-Romagosa et al. [70] recruited 51 stroke patients with upper-limb hemiplegia for 25 rounds of MI-BCI treatment. The Stroop color-word test (SCWT) and MCA were used to evaluate cognitive function before and after treatment, and there were no significant differences in memory and thinking scores, or scores on a self-reported questionnaire.

## 5. Safety and Stability of BCI

### 5.1. Signal Security

Sebastián-Romagosa et al. [70] tested the safety and availability of the BCI system in healthy elderly people using a memory training game. They reported no adverse events in any participants during any of the sessions. Immediate memory (*P* = 0.038), visuospatial/structure (*P* = 0.014), attention (*P* = 0.039), and delayed memory (*P* < 0.001) scores were significantly improved. Another BCI training designed to improve cognitive performance found that 10 participants (30.3%) reported a total of 16 adverse events, but all of them were “mild” (except for 1 “moderate” adverse event [105]). Overall, security and usability measures are high, and no serious adverse events have been reported when BCI is used in stroke rehabilitation. However, common treatment-related side effects such as transient nausea, fatigue, and headache may occur. Therefore, there is still a long way to go before BCI technology can really be applied on a large scale.

### 5.2. Signal Stability

Due to different BCI signal sources, BCI signal stability differs, but it can also be used to evaluate signal stability. Any information gathered in the first few hours of a single unit spike is considered erratic. Multiunit spikes (MSPs) are more stable and last longer than single unit peaks. Bionic BCIs that use MSPs can provide stable performance for about 6 months without recalibration, while bionic BCIs using LFPs remain stable for over a year [122]. Another study showed that MSP-BCI performance remained stable for up to 22 months in one monkey but was only stable for several weeks in another monkey [123], which may also point to individual heterogeneity in BCI application.

Milekovic et al. [124] found that LFP-BCI communication in a brainstem stroke-induced lockout syndrome and in a quadriplegic patients who had amyotrophic lateral sclerosis (ALS) was stable for 76 and 138 days without recalibration, respectively. BCI spelling rates of 3.07 and 6.88 correct characters per minute allow participants to type and write emails. Patients with locked-in syndrome can communicate daily using LFP-BCI without the need for intervention by a technician or caregiver. Quadriplegic patients were treated with repeated intracortical BCI for up to four and a half months. The method uses local field potentials (LFPs), which are more stable than neuronal action potentials, to decode the commands of the participants.

Natural environmental factors also have an impact on BCI signal stability. İşcan and Nikulin [125] examined many factors (i.e., psychology, speech, and audio interference) that might influence signal stability and the ability of patients to finish designated tasks. The experiment involved four conditions: the control group (which had no interference), the speaking group (who were instructed to loudly count from one to ten), the thinking group (who counted from one to ten in their head), and the listening group (who listened to someone else counting from one to ten). The results showed that the average classification accuracy for the speech and thinking groups decreased slightly, while the average classification accuracy of the hearing and the control group was not significantly different. The results indicated that decreases in BCI performance were related to changes in EEG signal quality and increased cognitive load, suggesting signal stability depends on many factors.

## 6. Difficulties and Challenges

There are several difficulties in the application of BCI in PSCI: (1) improving signal processing algorithms, exploring neural active patterns, quickly and accurately identifying task-related EEG signals, and eliminating interfering EEG signals are the most challenging tasks for the application of BCI systems in stroke-related cognitive impairment. (2) BCI needs to be adaptive to gender-based differences, needs to avoid differences in EEG signals, and needs to be calibrated to subject-specific needs. (3) The efficiency of BCI needs to be improved. (4) The development of a noninvasive, low-cost, easy-to-install BCI system suitable for stroke patients is also critical. In conclusion, BCI technology appears to enhance existing treatments for cognitive impairment after stroke. At present, BCI technology is developing rapidly, but there is still a long way to go before BCI is more widely applied.

## 7. Summary and Prospects

Cognitive decline after stroke is a major problem. Up to 30% of patients may develop dementia within three months after the occurrence of a cerebrovascular event. If TIA can be applied early and timely, then intervention against cognitive decline in stroke patients can be implemented, and patient prognosis can be improved. The application of BCI technology in poststroke cognitive impairment is a new direction for neurorehabilitation and has already been used in the assessment, training, rehabilitation, and treatment of PSCI. Studies have shown that the BCI can help improve PSCI. BCI can identify neuronal activity, classify and extract information, decode the subjects' intention, through NF and MI and repeated training, promote interneuronal interactions, change synapse potentials, improve brain compliance, improve brain network functional connectivity, adjust the balance between the hemispheres, promote neural plasticity-induced cortical reorganization, and improve cognitive function. Although BCI has shown some improvements for PSCI patients, more studies need to be carried out. Most current studies mainly focus on small samples and short-term observations of efficacy, and there is still a lack of large-scale randomized controlled trials that could verify its effectiveness and long-term efficacy. In addition, animal models cannot fully reflect the complexity of human cognition, which makes the project more challenging. However, with the continuous maturity of modern medical equipment and other technologies, and the application of hybrid BCI that combines multiple modes, BCI will become an even more practical and powerful way to treat PSCI in the future.

## Figures and Tables

**Table 1:** Comparative analysis of various methods used for recording features.

Risky	Signal source		Advantages	Disadvantages	Frequency of utilization	Description
Noninvasive	EEG	EEG signals	Cheap; high time resolution; portable	Low spatial resolution; longer training time; easily disturbed	Commonly used method	Measuring electrical signals produced by the human brain
		Spontaneous signals	Without external stimulus	Longer training time	Commonly used method	No external stimulus produced the signal
		Evoked signals (P300)	Price acceptable; training time short	Long time attention; easy to fatigue	Commonly used method	EEG signals are generated when stimulated by a flash of light/a latency of 250-500 ms
		Evoked signals (SSVEP)				EEG signals are generated when stimulated by looking at a frequency of flickering
	MEG		High spatial resolution; training time short; easy to record	Expensive; poor portability	Rarely used method	Record magnetoencephalogram signals generated by the human brain
	fMRI		High spatial resolution	Expensive; limited position; unportable low time resolution; unsuitable for real-time BCI; restricted to bold signals	Infrequently used method	Record the signals generated by brain metabolism
	fNIRS		Price affordable; high spatial resolution; unnecessary use precise parameter setting; portable	Low time resolution;	Infrequently used method	Record the signals generated by brain metabolism
	PET		Real time	Relatively high prices; complexity of the accompanying infrastructure	Only used in research and clinical neurology	Noninvasive measurement of cerebral blood flow, metabolism, and receptor binding in the brain

Invasive	ECoG		No training; high SNR; higher spatial resolution	Time consuming; easy induce epilepsy	Rarely used method	Electrodes are placed under the skull to measure electrical activity
	LFPs		High temporal and spatial resolution; high SNR	Easy lose signals	Rarely used method	Electrodes are placed under the skull to measure electrical activity

**Table 2:** Summary of articles on BCI-based applications for poststroke cognitive impairment.

Publications	Title	Signals	Sample	Tasks	Positive?
Yan et al. [81]	*Cognitive Alterations in Motor Imagery Process after Left Hemispheric Ischemic Stroke*	Event-related potential (ERP), event-related synchronization (ERD/ERS), P200, P300	11 ischemic stroke patients	Motor imagery (MI) training	Yes (cortical activation was altered differently in each cognitive substage of motor imagery)
Park et al. [82]	*Assessment of Cognitive Engagement in Stroke Patients from Single-Trial EEG during Motor Rehabilitation*	Electroencephalography (EEG); ERD	11 chronic stroke patients	Cognitive function assessment	Yes
Toppi et al. [78]	*Investigating the Effects of a Sensorimotor Rhythm-Based BCI Training on the Cortical Activity Elicited by Mental Imagery*	Sensorimotor (SMR)	2 hemisphere stroke patients	10 sessions SMR-based brain-computer interface- (BCI-) NF training	A: yes (spatial attention and memory) B: no
Cho et al. [64]	*The Effect of Neurofeedback on a Brain Wave and Visual Perception in Stroke: A Randomized Control Trial*	Electroencephalography (EEG)	27 stroke patients	Neurofeedback (NFB) training	Yes (concentration and visual perception)
Pichiorri et al. [83]	*Brain-Computer Interface Boosts Motor Imagery Practice during Stroke Recovery*	High-density electroencephalographic (EEG)	28 subacute stroke patients	BCI-supported-MI training	Yes (FMA score (*P* < 0.03). EEG sensorimotor power spectra occurred with greater involvement of the ipsilesional hemisphere in response to MI of the paralyzed trained hand)
Kober et al. [56]	*Specific Effects of EEG-Based Neurofeedback Training on Memory Functions in Poststroke Victims*	SMR, upper alpha	17 stroke patients	EEG-based neurofeedback training	70%: yes (verbal short- and long-term memory)
Reichert et al. [66]	*Shutting Down Sensorimotor Interferences after Stroke: A Proof-of-Principle SMR Neurofeedback Study*	Multichannel electroencephalography (EEG), sensorimotor rhythm (SMR)	1 stroke patient	10 sessions Sensorimotor rhythm (SMR) neurofeedback training	Yes (short- and long-term memory)
Kleih et al. [84]	*Toward a P300-Based Brain-Computer Interface for Aphasia Rehabilitation after Stroke: Presentation of Theoretical Considerations and a Pilot Feasibility Study*	P300, EPR	5 stroke patients	Visual-P300-based BCI spelling training	Yes (attention, accuracy in spelling, and reading)
Kober et al. [85]	*Upper Alpha-Based Neurofeedback Training in Chronic Stroke: Brain Plasticity Processes and Cognitive Effects*	Multichannel electroencephalogram (EEG)	2 chronic stroke patients	Upper alpha-based neurofeedback training	Yes (memory functions)
Tonin et al. [86]	*Behavioral and Cortical Effects during Attention-Driven Brain-Computer Interface Operations in Spatial Neglect: A Feasibility Case Study*	EEG	3 stroke patients	Covert visuospatial attention- (CVSA-) driven BCI training	Yes (visuospatial)
Lyukmanov et al. [82]	*Poststroke Rehabilitation Training with a Brain-Computer Interface: A Clinical and Neuropsychological Study*	Electroencephalography (EEG)	55 hemiplegic stroke patients	12 sessions BCI-supported mental training	Yes (the Taylor figure test, choice reaction test, head test, and online accuracy rate)
Shukin et al. [4]	*Poststroke Rehabilitation Training with a Brain-Computer Interface: A Clinical and Neuropsychological Study*	P300-evoked potentials	140 chronic cerebral ischemia patients	Neuropsychological testing	Yes
Kotov et al. [87]	*Usage of Brain-Computer Interface + Exoskeleton Technology as a Part of Complex Multimodal Stimulation in the Rehabilitation of Patients with Stroke*	Multimodal stimulation	44 stroke patients	Neural interface brain-computer + exoskeleton (BCI) training	Yes (memory, attention, visual, and constructive skills)
Foong et al. [88]	*Assessment of the Efficacy of EEG-Based MI-BCI with Visual Feedback and EEG Correlates of Mental Fatigue for Upper-Limb Stroke Rehabilitation*	EEG	11 stroke patients	EEG-based MI-BCI visual feedback training	Yes (fatigue-monitoring)
Chung et al. [89]	*Therapeutic Effects of Brain-Computer Interface-Controlled Functional Electrical Stimulation Training on Balance and Gait Performance for Stroke: A Pilot Randomized Controlled Trial*	Sensorimotor rhythm (SMR), midbeta, and theta	25chronic hemiparetic stroke patients	BCI-controlled functional electrical stimulation (BCI-FES) feedback training	Yes (executive capacity: gait velocity and cadence (*P* = 0.020), step length (*P* = 0.031))
Sebastián-Romagosa et al. [70]	*Brain-Computer Interface Treatment for Motor Rehabilitation of Upper Extremity of Stroke Patients—A Feasibility Study*	Electroencephalography signals	51 stroke patients	25 sessions MI-BCI training	No

## Data Availability

This article is a review article and does not contain relevant data.
